# High-throughput Bronchus-on-a-Chip system for modeling the human bronchus

**DOI:** 10.1038/s41598-024-77665-3

**Published:** 2024-11-01

**Authors:** Akina Mori, Marjolein Vermeer, Lenie J. van den Broek, Jeroen Heijmans, Arnaud Nicolas, Josse Bouwhuis, Todd Burton, Kazushi Matsumura, Kazuhiro Ohashi, Shigeaki Ito, Bart Kramer

**Affiliations:** 1 Scientific Product Assessment Center, Japan Tobacco Inc, 6-2, Umegaoka, Aoba-Ku, Yokohama, Kanagawa 227-8512 Japan; 2 Mimetas BV, De Limes 7 2342DH, Oegstgeest, The Netherlands

**Keywords:** Airway-on-a-chip, Organotypic culture, 3D-reconstructed airway epithelial cells, Lab-on-a-chip, Respiratory tract diseases, Chronic inflammation, Tissue engineering

## Abstract

Airway inflammation, a protective response in the human body, can disrupt normal organ function when chronic, as seen in chronic obstructive pulmonary disease (COPD) and asthma. Chronic bronchitis induces goblet cell hyperplasia and metaplasia, obstructing airflow. Traditional animal testing is often replaced by in vitro three-dimensional cultures of human epithelial cells to assess chronic cell responses. However, these cells are cultured horizontally, differing from the tubular structure of the human airway and failing to accurately reproduce airway stenosis. To address this, we developed the Bronchus-on-a-Chip (BoC) system. The BoC uses a novel microfluidic design in a standard laboratory plate, embedding 62 chips in one plate. Human bronchial epithelial cells were cultured against a collagen extracellular matrix for up to 35 days. Characterization included barrier integrity assays, microscopy, and histological examination. Cells successfully cultured in a tubular structure, with the apical side air-lifted. Epithelial cells differentiated into basal, ciliated, and secretory cells, mimicking human bronchial epithelium. Upon exposure to inducers of goblet cell hyperplasia and metaplasia, the BoC system showed mucus hyperproduction, replicating chronic epithelial responses. This BoC system enhances in vitro testing for bronchial inflammation, providing a more human-relevant and high-throughput method.

**Supplementary Information:**

The online version contains supplementary material available at 10.1038/s41598-024-77665-3.

## Introduction

The human respiratory tract comprises two main parts: the upper and lower respiratory tract. The nasal cavity, pharynx, and larynx belong to the upper respiratory tract, which primarily functions as air conductors. The trachea, bronchi, and lungs belong to the lower respiratory tract, which functions as the first line of defense against airborne materials^1^. A critical defense mechanism is the barrier function, which is achieved by tight and gap junctions in the epithelium, physically preventing airborne materials from invading the subepithelial compartment. In addition, the tracheal and bronchial epithelia play crucial roles in mucociliary clearance. The tracheobronchial epithelium comprised of basal, ciliated, and secretory cells. Ciliated cells express hair-like structures (cilia) on their surface, and secretory cells (including goblet and club cells) secrete mucus. Mucus captures inhaled airborne materials, which are cleared out of the respiratory tract by the beating cilia of ciliated cells^2,3^. However, once these defense mechanisms are impaired, inhaled materials can reach cellular compartments and trigger various cellular responses, including inflammation.

Airway inflammation is a protective response of the body to airborne materials, such as toxins, pollutants, irritants, and allergens, and is normally a temporal biological event. However, when inflammation becomes severe and chronic, it can disrupt normal organ function, leading to conditions such as chronic obstructive pulmonary disease and asthma^4,5^.

The mechanisms underlying chronic airway inflammation are complex, and its symptoms often manifest at multiple sites within the airway^6,7^. In the bronchial region, chronic inflammation in the airways often leads to bronchitis, during which bronchial tissues exhibit abnormal cell populations and impaired mucociliary clearance function. These results are due to inflammation-induced hyperplasia and metaplasia of goblet cells, leading to hyperproduction of mucus and thickening of epithelial cell layers. As a result, patients with chronic bronchitis often experience difficulty breathing due to airflow obstruction caused by mucus hyperproduction and airway remodeling^8–10^.

Inhalation animal testing has traditionally been used for modeling airway diseases and evaluating the effects of tobacco products, chemicals, pesticides, and pharmaceuticals^11–13^. However, this approach has limitations in predicting its effects in humans, and the growing call for animal welfare has driven the development of alternative test method^14^.

Two culture methods are commonly used for in vitro toxicological assessment and analysis of disease mechanisms in the human bronchi. The first is the two-dimensional culture system, which is a simple and low-cost culture method, in which airway epithelial cells are cultured in flasks or plates as monolayers. This system is mainly used for acute cell response analyses, such as cytotoxicity and cytokine production, and is useful for screening purposes in toxicological and pharmacological evaluations. However, this can lead to misinterpretation because the cells lack polarization and functionality, and thus do not effectively represent airway cells in vivo.

**Figure 1:**
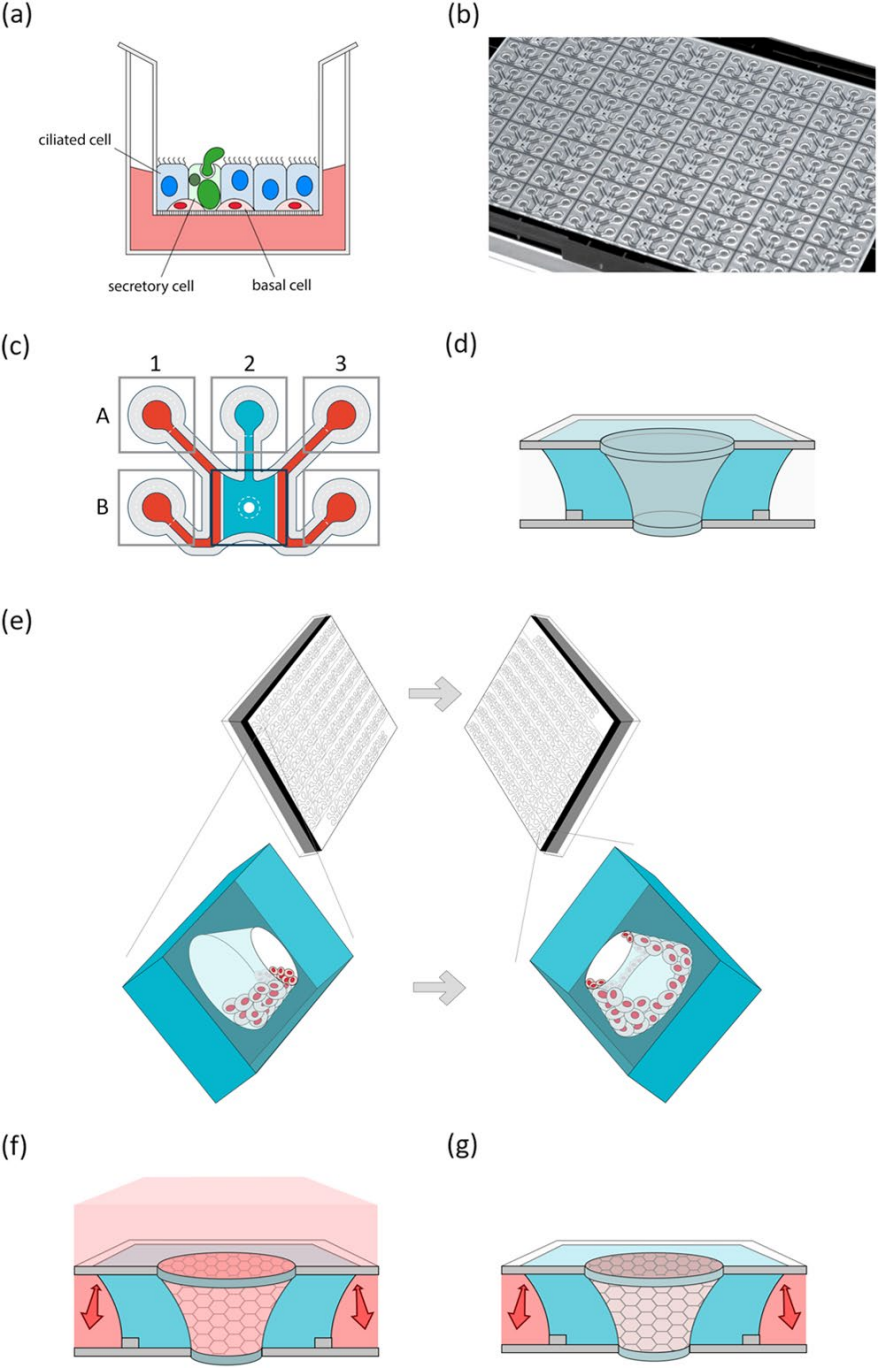
The microfluidic air exposure system OrganoPlate Air. (**a**) A schematic image of a three-dimensional culture system. The cell layers consist of basal, ciliated, and goblet cells on the membrane in the insert. The apical side is exposed to air. (**b**) A photograph of the bottom of the OrganoPlate Air, showing microfluidic chips based on a standard 384-well titer plate format. (**c**) A schematic overview of the chip layout in the OrganoPlate Air, comprising six wells connected by microfluidic channels. Perfusion channel inlets are in A1 and A3, perfusion channel outlets are in B1 and B3, the Gel Inlet is in A2, and the central chamber is in B2. Extracellular matrix (ECM; blue) is loaded into the chip through the gel inlet, and the surface tension of the loaded ECM at the edges (arrowheads) prevents flowing of the ECM into the center hole, leaving the central cylinder in the central chamber empty. The cells can be seeded in this cylindrical lumen, forming a tubular structure. During culture, medium (red) can be added to the perfusion channels and to the central chamber for submerged culture (pink). The air-liquid interface can be initiated by removing medium from the central chamber. The perfusion channels supply the culture with medium, via nutrient diffusion through the ECM in the central chamber.

Conversely, a three-dimensional (3D) culture system is a more sophisticated approach that mimics the tracheobronchial epithelium more accurately (Fig. 1(a)). Such 3D cultures contain functional epithelium with pseudostratified columnar and multitype cells, including basal cells at the bottom of the layer, ciliated cells, and goblet cells containing mucus^15,16^. In addition, 3D-cultured airway epithelial cells are suitable for long-term culture, which enables the induction of chronic states. For example, increased epidermal growth factor (EGF) levels and continuous treatment with interleukin (IL)-13 can lead to goblet cell hyperplasia and metaplasia in 3D human bronchial epithelial cells (HBECs)^17,18^. The result of 3D HBECs show promise for mimicking chronic bronchial responses. However, considering the actual human bronchial structure, such cultures have room for improvement.

Most of the current structure of 3D HBECs is a horizontal plane, which differs from the physiological tubular structure of human bronchi. Considering the effect of surrounding cell populations on cell growth, the tubular structure of HBEC cultures would be more representative of the human bronchial system. In addition, considering airborne material exposure through human inhalation, modeling a tubular structure would be more biologically relevant because inhaled substances are normally exposed to airway epithelial cells in the tubular lumen. Furthermore, stenosis and airflow obstruction of chronic bronchitis are not reproduced in a planar culturing method but can be reproduced in a tubular structure. Development of a culture method that mimics such a physiological environment could be beneficial for developing more predictive test methods.

Recently, physiologically relevant in vitro culture methods have been progressively developed, and they are anticipated to serve as an alternative for animal testing. Organ-on-a-Chip (OoC) technology has emerged and mimics the physiological environment in thumb-size, or smaller, microfluidic chips^19,20^. They can be broadly classified into high-throughput and high-function models. High-throughput OoCs typically use systems that are compatible with standard labware, such as multi-channel pipettes and robotics. They allow for conducting experiments with many replicates and conditions^21^. However, to achieve high-throughput capability, these chips may sacrifice some functionality, such as organ structure and dynamics. Conversely, high-function OoCs are designed to mimic organ-specific structure or dynamics by recreating the spatial environment of cells and by incorporating special devices that realize in vivo conditions, e.g., organ substructure, motion, and blood flow, at the cost of throughput. In high-function OoCs, the dynamics of the cells and material being tested are well reproduced^22,23^.

The development of Airway-on-a-Chip technology has mainly focused on alveolar models^24^, resulting in a limited number of reported tracheal and bronchial models. Benam et al.^25^ reported a ‘small airway-on-a-chip’, which incorporates functional airway epithelium and is capable of mimicking breathing motion, air, and blood flow. This model demonstrated potential for studying airway inflammation. Similar models have also been reported, as summarized by Lagowala et al.^26^. Moreover, Park et al. reported “E-FLOAT” which enables on-chip as well as off-chip analyses under physiological airflow^27^, and Wuyang et al. recently reported that tubular airway-on-chip model which enables to investigate ventilation dynamics^28^. Thus, advancements in Airway-on-a-Chip technology offer opportunities for selecting an appropriate in vitro model based on research objectives. Nevertheless, the throughput of these models is generally low, and improving throughput while maintaining high physiological relevance remains a challenging issue.

In this study, we developed Bronchus-on-a-Chip (BoC) with both tubular structured functional epithelial cells and high-throughput capabilities on a specialized plate, the OrganoPlate Air. This tubular structured culture system represents a human-relevant environment for aerosol exposure inside a tube, mimicking the actual route of exposure. We believe that this hybrid approach, combining high functionality and high throughput performance in the BoC, will be valuable for studying airway inflammation and chronic-phase biological responses that reflect the complexity of in vivo situations in humans. Additionally, the model offers expanded options for the fit-for-purpose use of airway-on-a-chip models.

## Materials and methods

### Cell culture

HBECs (Lonza, CC-2540, lot 623950) were expanded in T75 culture flasks (ThermoFisher, 156499) with PneumaCult ExPlus medium (StemCell Technologies, 05040) containing 1% penicillin/streptomycin (Sigma, P4333). Medium aliquots were supplemented with hydrocortisone (StemCell Technologies, 07925) according to the manufacturer’s instructions Cells were detached with trypsin/ethylenediaminetetraacetic acid (Lonza, CC-5012) for 5 min and neutralized with Trypsin Neutralization Solution (Lonza, CC-5002). Cells were frozen in passage 2 in ExPlus medium with 40% fetal bovine serum (Gibco, 16140-071) and 10% dimethyl sulfoxide (Sigma, D8418) and stored at − 150 °C. To initiate an experiment, HBECs were thawed in ExPlus medium and directly seeded into OrganoPlate Air (see next section). After the cells formed a confluent layer, the medium was switched to PneumaCult ALI medium (differentiation medium, StemCell Technologies, 05001) containing 1% penicillin/streptomycin. Medium aliquots were supplemented with Heparin Solution (StemCell Technologies, 07925) and hydrocortisone according to the manufacturer’s instructions and were used within 2 weeks. Immediately prior to medium change with differentiation medium, 250 nM matrix metalloproteinases-inhibitor GM6001 (Abcam, ab120845) was added to avoid degradation of the collagen scaffold.

### OrganoPlate air

HBEC cultures were established in a newly developed plate, the OrganoPlate Air (Mimetas BV), which consists of 62 microfluidic chips patterned underneath a 384-well titer plate (Fig. 1(b)). Each chip connects six wells that contain two perfusion channels and a gel channel leading towards a central chamber (Fig. 1(c); well B2). The design of the novel OrganoPlate Air is adapted from the OrganoPlate Graft (Mimetas BV)^29^, with an opening at both the top and bottom glass layer of the central chamber. To maintain sterility, a bottom cover was placed underneath the plate. The PhaseGuides present on either side of the central chamber prevent the liquid extracellular matrix (ECM) from overflowing into the adjacent channels before it solidifies^30^, omitting the need for artificial membranes. In this manner, the adjacent perfusion channels can be used for perfusion and diffusion of nutrients through the ECM in the central chamber. In a similar manner, the surface tension of the openings in the glass at the top and bottom of the central chamber also prevent the liquid ECM from overflowing into the opening itself, leaving a tapered tubular cavity from top to bottom within the ECM filled central chamber after it solidifies. In this cavity, cells can be cultured in a tubular fashion, leaving full access to the apical surface.

Bovine collagen (5 mg/mL; PureCol EZ, Advanced BioMatrix, 5074) was used as an ECM inside the central chamber. PureCol EZ (3 µL) was loaded to the gel inlet (Fig. 1(c); well A2) to fill the chamber using an electronic single channel pipette (Sartorius, Picus). After ECM loading, the plate was placed at 37 °C for 40 min, and then 50 µL of phosphate-buffered saline (PBS) (Gibco, 70013065) was added to the gel inlet to keep the ECM hydrated. After 1 day at 37 °C, 1 µL of PBS was added to all perfusion channel inlets (Fig. 1(c); wells A1 and A3), and the plates were incubated at 37 °C for at least 6 days which increases the stiffness of the ECM. Then the chip is ready for cell seeding within the open cylinder structure in the ECM (Fig. 1(d)).

Immediately prior to cell seeding, ExPlus medium was added to the perfusion channels using an electronic 8-channel pipette (Sartorius, Picus) (50 µL into each perfusion inlet and outlet; Fig. 1(c); wells A1, A3, B1, and B3). Cells were seeded inside the cavity in the central chamber by pipetting 0.25 µL of a 20 000 cells/µL cell suspension, resulting in 5 000 cells/chip. The surface tension at the bottom of the cavity facilitated cells or medium from remaining within the cavity. The plate was incubated at 37 °C for 4 h on one of the long sides of the plate and then switched to the opposite long side of the plate overnight to allow the cells to settle all around the gel (Fig. 1e). After cell attachment, 50 µL of ExPlus medium was carefully added to the central chamber (Fig. 1(c); well B2), and the plate was placed on a rocking platform (OrganoFlow, Mimetas BV) set at a 14° inclination with an 8-min interval to initiate submerged cell culture with bidirectional flow through the perfusion channels (Fig. 1(f). After 3 days of culture, a confluent cell layer of epithelium was formed growing within the cavity against the ECM; the medium in the perfusion channels was changed to differentiation medium, and the medium in the central chamber was replaced with Hanks’ Balanced Salt Solution (HBSS, Sigma H6648).

On day 10 after cell seeding, air-liquid interface culture was initiated and maintained until the end. Culture medium is supplied through the perfusion channels throughout the experiment. To achieve this air-liquid interface culture, the liquid in the central chamber and perfusion channels was removed, and then the bottom cover was removed. The plate was then inverted and left to air dry for approximately 3 min. Next, the bottom cover was placed back on the plate, medium was added to the perfusion channel inlets and outlets as described before, and culture was continued with an air-liquid interface present (Fig. 1(g)).

### Cilia assessment

From day 14 onwards, the presence of cilia was assessed. To increase the visibility of the air-liquid interface cultures, 50 µL of HBSS was added to the central chamber. The number of beating cilia per chip was observed with an optical microscope and categorized from 0 to 3: 0 indicates absence of cilia, 1 indicates small patches of cilia, 2 indicates larger patches of cilia, and 3 indicates large areas of cilia. Representative videos of categories 1 to 3 are supplied in supplementary video 1(b). After the assessment, the air-liquid interface was re-initiated as previously stated. Statistics were performed with the ‘one-way ANOVA’ function in GraphPad Prism version 10.

### Immunohistochemistry

HBEC cultures were fixed with 3.7% formaldehyde (Sigma, 252549) in HBSS for 10 min, washed once with PBS for 5 min, and stored with 50 µL of PBS per well at 4 °C until immunofluorescent staining.

Fixed HBEC cultures were simultaneously permeabilized and blocked for 45 min with 2% fetal calf serum (FCS, Gibco, A13450), 2% bovine serum albumin (Sigma, A2153), and 0.3% Triton X-100 (Sigma, T8787) in PBS. Afterwards, the cultures were incubated overnight with primary antibodies in antibody solution containing 2% FCS + 2% bovine serum albumin + 0.1% Triton X-100 in PBS, adding 20 µL to all wells except gel inlet After a 5 min wash with 4% FCS in PBS, the cultures were incubated for 1–2 h with secondary antibodies in antibody solution, including Sytoxgreen (1:5 000; ThermoFisher, S7020) as a nuclear stain. After the incubation, the cultures were washed once with PBS, and then 30 µL CUBIC-R+ (TCI, T3741) was added to all wells, except the gel inlet, at least 4 h prior to imaging. All the steps were performed at room temperature. The following primary antibodies were used: rabbit anti-human cytokeratin 5 (1:100; Abcam, ab52635), mouse anti-human βIV tubulin (1:100; Sigma, T7941), mouse anti-human mucin (MUC) 5AC (1:100; ThermoFisher, MA512178), and rabbit anti-human ZO-1 (1:100; ThermoFisher, 61-7300). The following secondary antibodies were used: donkey anti-mouse AF555 (ThermoFisher, A31570), goat anti-mouse AF555 (ThermoFisher, A21422), goat anti-rabbit AF488 (ThermoFisher, A11008), and donkey anti-rabbit AF647 (Sigma, SAB4600177), all at a dilution of 1:250. Immunofluorescent images were captured with an ImageXPress XLS-C HCI system (Molecular Devices) at 10× magnification, acquiring z-stacks with a distance of 5 μm. The maximum projection images were used for visual representation. The sum projection images were analyzed and quantified in FIJI, the ‘measure integrated density’ function was used for quantification of the marker expression of the area captured during imaging. The ‘analyze particles’ function was used for quantification of the number of nuclei. Statistics were performed with the ‘unpaired one-way ANOVA’ function in GraphPad Prism version 10. The color coding of the nuclear z-location was done as post-image analysis by applying a color gradient to the z-location of the nucleus signal using Fiji through “Temporal-color code” function.

### Barrier integrity assay

Next, the barrier integrity of HBECs cultured on the OrganoPlate Air was assessed. For this, 0.5 mg/mL FITC-Dextran (150 kDa; Sigma, 46946) in PBS was added to the apical side of the cell layer, and its diffusion to the basal side of the cell layer was measured. HBSS was aspirated from the central chamber if present, and 50 µL of FITC-Dextran was added to the central chamber and incubated for 15 min at 37 °C after which it was aspirated. The central chamber was washed once with PBS and aspirated prior to acquiring images with an ImageXPress XLS Micro HCI System (Molecular Devices) at 4× magnification. The fluorescence intensity of the ECM in the central chamber was quantified in FIJI, excluding the area of the center hole (Fig. 4(a)). Statistics were performed with the ‘unpaired two-tailed t-test’ function in GraphPad Prism version 10.

### Exposure

On day 21 of culture, differentiated HBEC cultures were exposed to EGF (PeproTech, AF-100-15) for 7 days by adding 1 or 5 ng/mL EGF to the differentiation medium in the perfusion channels. EGF was re-added with every medium change. On day 28, the cultures were exposed to 0.5, 1, or 5 ng/mL IL-13 (PeproTech, 200 − 013) for 7 days in a similar manner (Fig. 5(a)). Both exposures were performed during air-liquid interface culture, and no medium or HBSS was present in the central chamber.

## Results

### The OrganoPlate air enables tubular cell cultures

The OrganoPlate Air is a novel microfluidic device consisting of 62 chips patterned underneath a standard 384-well titer plate, enabling perfusion and nutrient diffusion through a central chamber filled with an ECM (Fig. 1(b) and (c)). It enables both submerged and air-liquid interface cell cultures with access to the culture medium or air on the apical surface, respectively. In this study, we used the OrganoPlate Air to culture HBECs in a tubular fashion against a collagen ECM.

**Figure 2:**
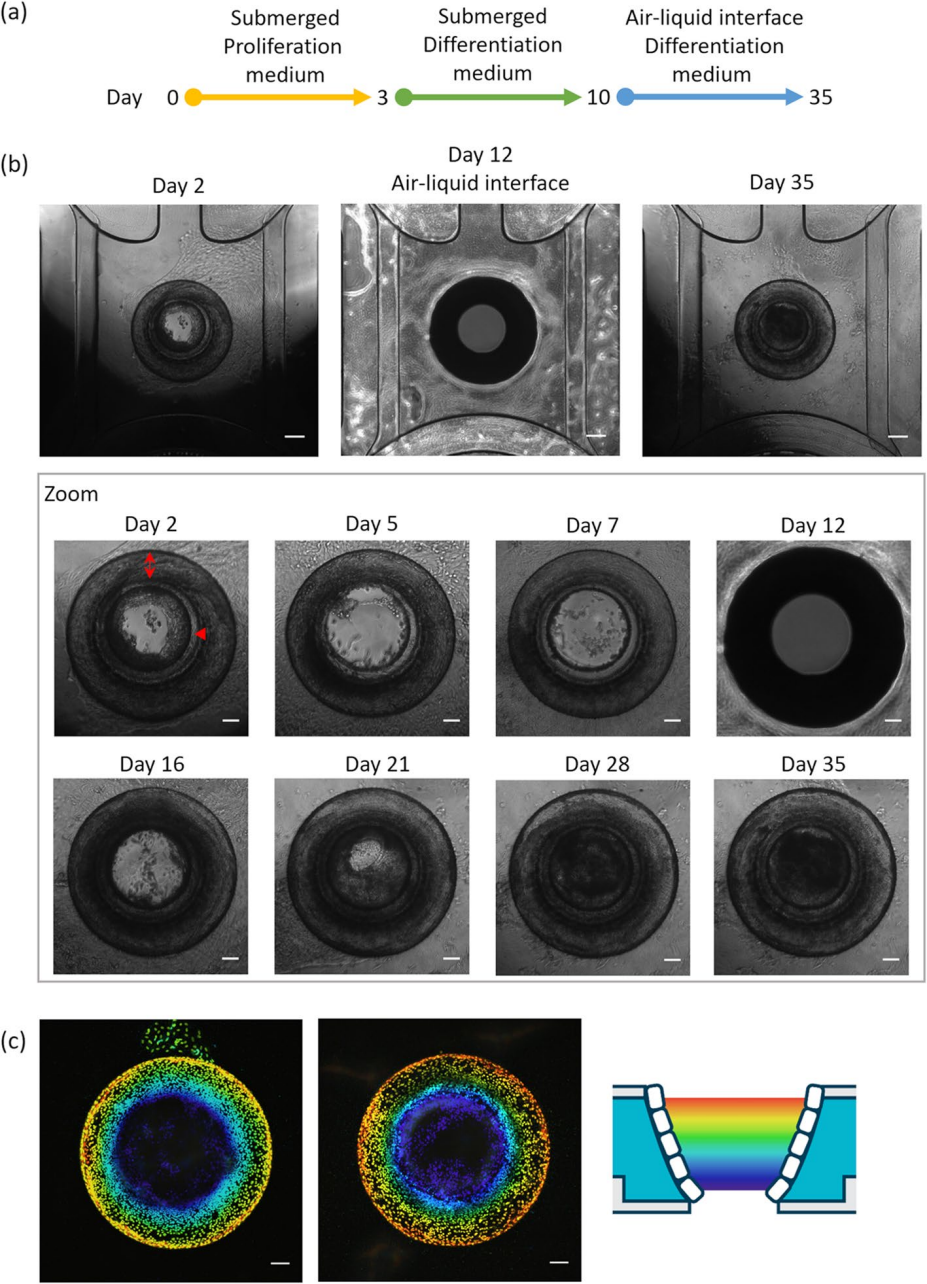
Bronchus-on-a-Chip culture in the OrganoPlate Air. (**a**) The experimental timeline of bronchial epithelial cell cultures in the OrganoPlate Air. Cells are seeded in the OrganoPlate Air on day 0, switched to differentiation medium on day 3, and air-liquid interface culture is initiated on day 10. Cultures are maintained up to day 35. (**b**) Phase-contrast images of cultures over time. For regular imaging, Hanks’ Balanced Salt Solution (HBSS) was added to the central chamber prior to imaging to improve visibility of the culture. Double arrow and arrow head show gel surface and bottom glass respectively. The image on day 12 was taken without HBSS in the central chamber. Scalebars = 200 µm and 100 µm for magnified images. (**c**) Nuclear staining after fixation on day 35 with color coding for the z location within the chip, as indicated by the schematic. Scalebar = 100 µm.

After cell seeding, the cells formed a confluent monolayer within the cavity against the ECM by day 3 (Fig. 2(a) and (b)). The cell layer remained confluent throughout the culture period (magnified images in Fig. 2(b)). On day 10, the culture was transitioned to an air-liquid interface setup, allowing direct exposure of the cells to air for the remaining duration of the culture. This resulted in a shadow being cast on the area against the ECM, thereby limiting visibility visual inspection and imaging (Fig. 2(b, day 12)). To mitigate this issue, HBSS was added to the central chamber immediately prior to observation to improve visibility. HBSS was subsequently removed to re-establish the air-liquid interface, and culturing was continued with cells directly exposed to air. To assess the confluency and tubular structure of the HBEC culture, Sytoxgreen nuclear fluorescent stain was used for visualization of the confluent cell layer. Processing of the acquired images with a color coding gradient for the z-location within the chip, confluency across the entire area against the ECM could be qualified (Fig. 2(c)). The complete area against the ECM was covered with cells, creating a confluent and stable tubular structure of bronchial epithelium. The bottom and top of the tubular structure remain open, allowing for unobstructed airflow through the chip.

### Bronchial epithelium cultured in the OrganoPlate air differentiates and forms a polarized tubular structure

**Figure 3:**
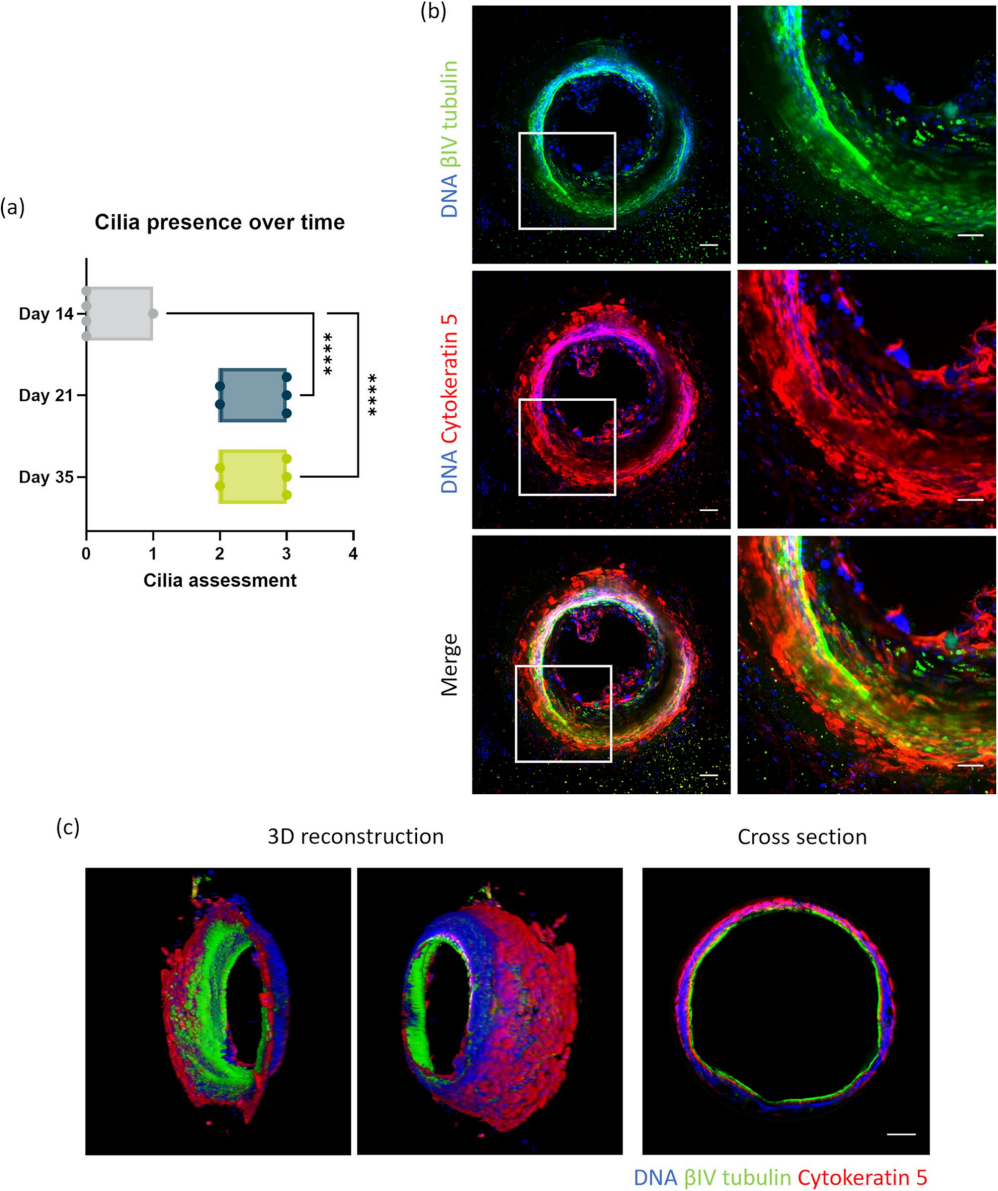
Differentiated and polarized tubular structure of Bronchus-on-a-Chip. (**a**) Visual cilia assessment on days 14, 21, and 35 of culture: 0 (no cilia), 1 (small patches), 2 (large patches), and 3 (large areas) categorized by microscopic observation; five replicates for all days. Mean with error bars± SD, statistical test – ordinary one-way ANOVA (**** *p* < 0.0001). (**b**) Immunofluorescence images of cells in Bronchus-on-a-Chip on day 35 of culture: 10× maximum projection images of βIV tubulin (green), cytokeratin 5 (red), and DNA (blue) in the left images and a magnification of the framed area in the right images. Scalebars = 100 µm (left images) and 50 µm (right images). (**c**) Three-dimensional reconstruction images and a cross-section of Bronchus-on-a-Chip showing a polarized tubular structure: βIV tubulin (green), cytokeratin 5 (red), and DNA (blue). Scalebar = 100 µm.

For the differentiation of HBECs into a polarized and ciliated epithelium, the medium was switched from expansion medium to differentiation medium, which was applied to the basal side of cells. Subsequently, the apical side was cultured in an air-liquid interface environment. On days 14, 22, and 36 of culture, cilia were visually categorized as follows: no cilia (0), small patches (1), large patches (2), or large areas (3) (Fig. 3(a)). A video of beating cilia on day 21 of culture can be found in supplementary video 1(a), as well as a representative video of categories 1 to 3 in supplementary video 1(b). An evident increase in the abundance of cilia was observed: the average score was 0.2 on day 14 and 2.7 on day 21, which remained almost consistent up to day 35.

In addition to the visual assessment of cilia, cultures were fixed on day 35 and stained for cytokeratin 5 (basal cells) and βIV-tubulin (cilia) to confirm HBEC differentiation. Maximum projection images showed consistent cytokeratin 5 expression in the BoC. The presence of cilia was also revealed by βIV-tubulin staining, which was observed in cultures in either large areas or small patches (Fig. 3(b)). To enhance visualization of the tubular structure of the cultures, a 3D reconstruction was generated from the immunofluorescent staining of nuclei, cytokeratin 5, and βIV-tubulin. This demonstrated the confluency around the area against the ECM (Fig. 3(c) and supplementary video 2). Furthermore, it illustrated that the basal cells were located at the basal side against the ECM, whereas cilia were present at the apical side facing the lumen. This morphology was further confirmed in a cross-section (Fig. 3(c)) with the basal cells and cilia present at the basal side and inside of the tubular structure, respectively.

### Cells in the BoC display barrier formation

**Figure 4:**
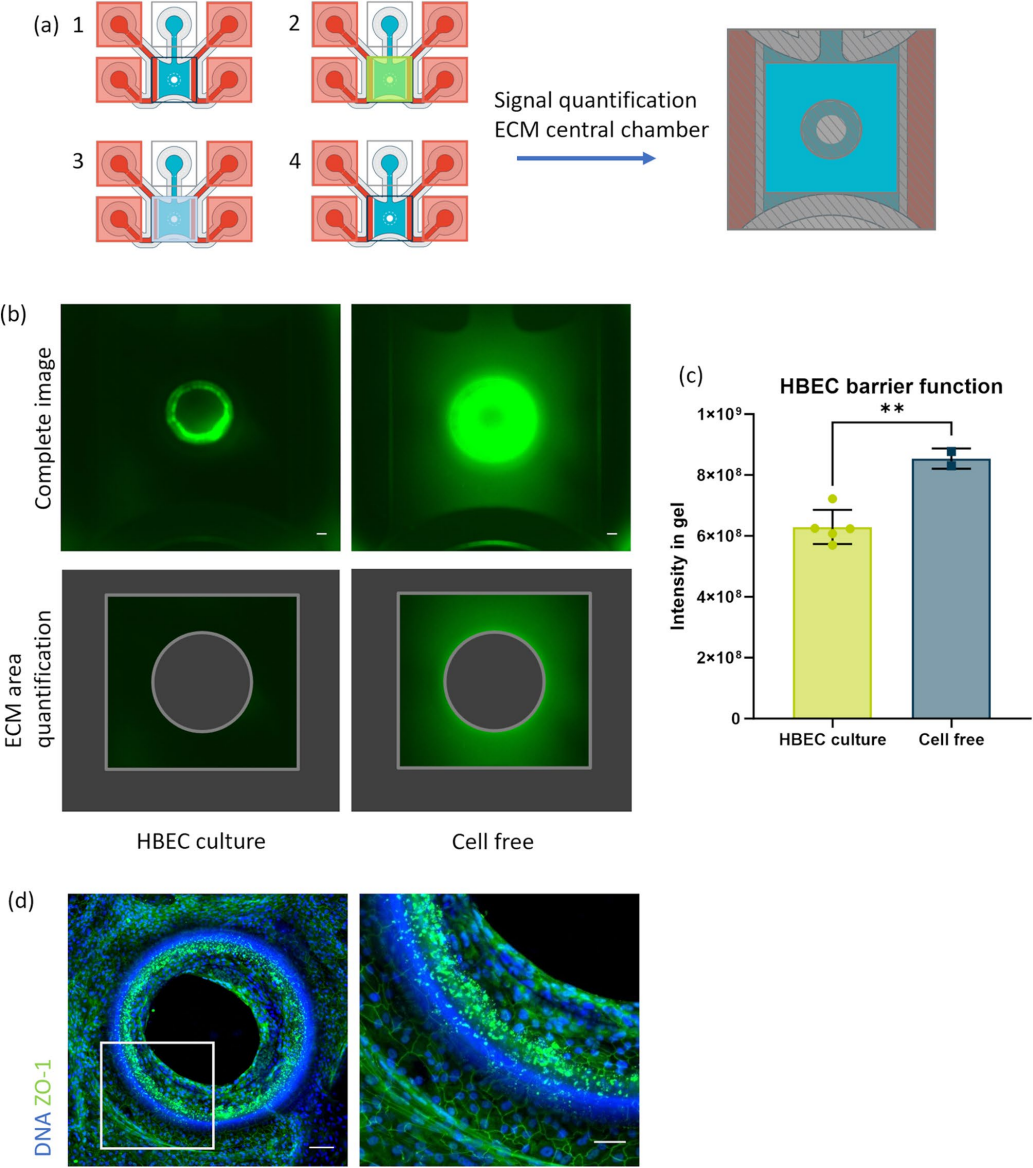
Assessment of barrier function in Bronchus-on-a-Chip. (**a**) A schematic overview of the barrier integrity assay. Any liquid present in the central chamber is removed (1), fluorescent solution is added to the central chamber (2) and incubated for 15 min prior to a wash (3), and removal of liquid in the central chamber before imaging (4). The extracellular matrix (ECM) area is depicted for signal quantification, excluding the gray area from analysis. (**b**) Barrier integrity assay images of chips with and without cells on day 21 after applying 150 kDa FITC-Dextran. Scalebar = 100 μm. (**c**) Quantification of barrier integrity assay by measuring fluorescent intensity of the ECM area of the Bronchus-on-a-Chip, indicated in (a) and (**b**) as the area between the gray frames. Mean with error bars ± SD, chips with cells (*n*  = 5); cell-free chips (*n*  = 2). Statistical test – unpaired two-tailed T-test (** *p*  < 0.01). (**d**) Fluorescence images Bronchus-on-a-chip, 10x maximum projection images of ZO-1 (green) and DNA (blue) in the left image and a magnification of the framed area in the right image. Scalebars = 100 μm (left image) and 50 μm (right image).

To evaluate the barrier formation and function of cells in the BoC, a barrier integrity assay was performed on day 21 of culture. When cells have leak-tight barrier function, they inhibit dextran diffusion from center hole (Fig. 4(a)). In wells without cells and barrier function as a control, the diffusion of a fluorescently labeled dextran molecule was detected in the ECM. Conversely, a functional cell barrier was observed as a lack of fluorescent signal in the ECM area of the central chamber on day 21 (Fig. 4(b)).

Quantification of the fluorescent signal in the ECM of the central chamber revealed a 35% higher fluorescence intensity in cell-free chips compared to BoCs (Fig. 4(c)). Presence of tight junctions at the cell borders was confirmed by ZO-1 staining (Fig. 4(d)), further indicating barrier function of the BoC.

### Compound exposure on BoCs leads to increased mucus production

**Figure 5:**
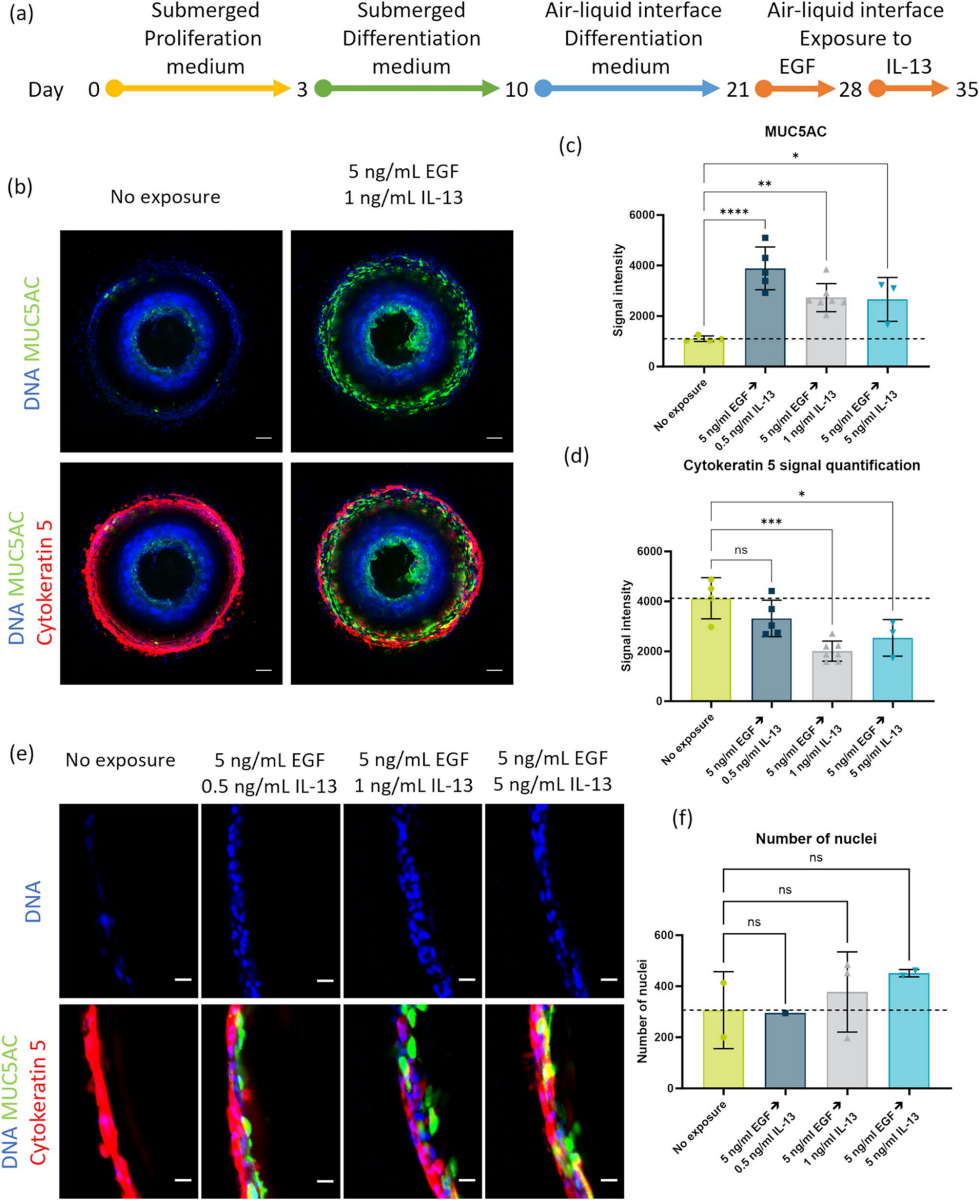
Increased goblet cell formation after epidermal growth factor (EGF) and interleukin (IL)-13 exposure of Bronchus-on-a-Chip . (**a**) Timeline of epithelial cell culture and exposure. Culture differentiation up to day 21, followed by 1-week exposure to EGF and 1-week exposure to IL-13. (**b**) Fluorescence images of Bronchus-on-a-Chip on day 35, after exposure to 5 ng/mL EGF followed by 1 ng/mL IL-13. 10× maximum projection images of MUC5AC (green), cytokeratin 5 (red), and DNA (blue). Scalebar = 100 μm. (**c**) Quantification of MUC5AC signal intensity after exposure to 5 ng/mL EGF and 0.5–5 ng/mL EGF (3–7 replicates). Mean with error bars ± SD. (**d**) Quantification of cytokeratin 5 signal intensity after exposure to 5 ng/mL EGF and 0.5–5 ng/mL EGF (3–7 replicates). Mean with error bars ± SD. (**e**) Magnified fluorescence images of Bronchus-on-a-Chip on day 35 after exposure to 5 ng/mL EGF and 0.5–5 ng/mL IL-13. Cross-section of area against gel, displaying MUC5AC (green), cytokeratin 5 (red), and DNA (blue). Scalebar = 20 μm. (**f**) Quantification of the number of nuclei in the complete cross-sections of which a magnified image is depicted in (**e**) (1–3 replicates). Mean with error bars ± SD. Statistical test – ordinary one-way ANOVA (ns = not significant, * *p*  < 0.05, ** *p*  < 0.01, *** *p* < 0.001 , **** *p* < 0.0001).

To assess the chronic response of BoC cells to compound exposure, cultures were exposed to EGF and IL-13, which are involved in airway inflammation and remodeling pathways. On day 21 (11 days after initiation of the air-liquid interface), cultures were exposed to EGF for 7 days, and then to IL-13 for 7 days, adding up to a culture time of 35 days (Fig. 5(a)). The air-liquid interface was maintained throughout the entire exposure period. The consecutive exposure to EGF and IL-13 led to an increase in mucus production as characterized by an increased signal for MUC5AC (Fig. 5(b)). This increased mucus production did not depend on IL-13 concentration (supplementary Fig. 1), which was confirmed with MUC5AC signal quantification. A 2.5–3.5-fold increase in signal intensity was detected after EGF and IL-13 exposures compared to the control condition (Fig. 5(c)). Conversely, the presence of basal cells displayed a 0.5–0.8-fold decrease after exposure to EGF and IL-13, as revealed by cytokeratin 5 signal quantification (Fig. 5(d)).

Another characteristic of airway inflammation and remodeling is thickening of the cell layer, which can be triggered through an inflammatory pathway^31^. Although methods for visualizing cross-sectioned BoC and quantifying the cell population require further improvement, we nevertheless observed that 2-week exposure to EGF and IL-13 resulted in an increase in the number of nuclei from cross-sectioned images (Fig. 5(e)). Despite no statistical difference, a trend of increasing numbers of nuclei with increasing IL-13 concentrations can be observed in the quantification of nuclei in the cross-sections (Fig. 5(f)).

## Discussion

The BoC was developed by combining a 384-well titer plate with a novel microfluidic system. The design enables the use of standard labware equipment and achieves high throughput with 62 chips/plate. This chip system belongs to the category of OoC systems with the highest throughput, which enables flexible study designs.

The characteristic structure of the OrganoPlate Air facilitates a tubular structural cell culture similar to that of bronchi, which is suitable for air exposures. To achieve this, we intentionally designed the opening (top side) hole to be larger than the end (bottom side) hole in the OrganoPlate Air, resulting in a gentle slope of the bronchial tubule. This slope enhances the visibility of the culture under microscopic examination during submerged culture. Upon initiating the air-liquid interface, the cells become invisible due to light refraction and scattering, indicating a successful air-liquid interface.

The bronchial tubule-on-a-chip demonstrated a leak-tight cell layer with an appropriate cell population, as indicated by the expression of β-tubulin IV and Cytokeratin 5 at the luminal surface and basement layer, respectively. The expression of these markers has been reported to identify ciliated cells and basal cells in the human bronchi^32–34^, as well as in 3D HBEC cultures. This suggests that our BoC model maintains an appropriate cell population with correct spatial organization. Mucus-producing cells (i.e., MUC5AC-positive cells) were rarely observed. To reproduce a disease-state, the cell population and its functionality should be similar to those of in vivo tissues because airway disorders are often associated with abnormal and dysfunctional tissue structures caused by aberrant cell differentiation^35,36^. In such disease-state airway tissues, impairment of both barrier and ciliary functions is generally observed^37,38^. Our BoC enabled the assessment of ciliary function and cell population alterations; therefore, we believe that it is useful for assessing disease risk. In addition, the BoC could be maintained for up to at least 35 days, which could be long enough to induce chronic effects of stimuli.

To demonstrate the effect of prolonged cytokine exposure in our BoC, we performed a proof-of-concept study involving prolonged exposure to EGF and IL-13. The regulation of the EGF receptor (EGFR) signaling pathway is pivotal for the maintaining differentiation and proliferation of airway epithelial cells^39,40^. However, aberrant EGFR activation causes anti-apoptotic effects and thus leads to abnormal basal cell proliferation^41^. Activation of the EGFR signaling pathway is also related to mucus production in airway epithelial cells via the specificity protein-1 (Sp1) axis^42^. In addition, IL-13 is a well-known inducer of goblet cell metaplasia and hyperplasia both in vitro and in vivo^43–45^. As a result of 2-week exposure to EGF and IL-13, we observed an expected increase in MUC5AC-positive cell numbers and a decrease in basal cell numbers. This suggests that basal cells turned into mucus-producing cells, resulting in increased mucus production. These results demonstrate the utility of BoC in chronic exposure studies and for inducing disease states. In addition, the BoC model is compatible with co-culture with other cell types. For example, fibroblasts and smooth muscle cells can be embedded in the ECM compartment, and immune cells can be added into medium flow. Such a co-culture system could further recapitulate the human relevance of the BoC and could thus be a natural future scope.

Traditionally, 3D-HBECs are used for exposure studies of airborne materials, and thus various exposure apparatuses are available for these culture methods^46–48^. However, most of them expose aerosols from top to bottom in a vertical manner. Consequently, the dynamics of aerosols cannot be fully reproduced with horizontally cultured cells. This approach can result in uneven aerosol deposition within a single sample^49^. In the human bronchi, aerosols travel from the mouth or nose to deep lungs through airway tubules. Previous studies showed shear stress alters cell characteristics such as protein expression, barrier function, and nano particle delivery^50,51^. Here, we achieved airway epithelial cell cultures with a tubular structure that closely mimics human airways. This opens up the possibility of reproducing exposure scenarios that are more relevant to humans. The air flow could provide shear stress to the HBECs in BoC, expecting manifestation of air-flow-associated functional characteristics such as coordinated cilia movement^52^. For this purpose, we are developing an aerosol exposure apparatus for horizontal 3D-HBECs which is capable of reproducing human-relevant airflow and exposure scenario. For instance, aerosol exposure apparatus for horizontal 3D-HBECs can adopt to BoC when we develop an attachment for BoC which allows to flow air and aerosol inside the BoC tubule, from the top to bottom.

We recognize that our BoC still has room for improvement. Handling BoCs can be technically challenging, and we sometimes experienced that the ECM sporadically failed to construct a robust culture interface. This could be due to secreted MMPs from HBECs^53^, therefore, we used MMP inhibitor (GM6001) to prevent biological degradation of ECM. However, MMPs play a critical role in inflammation and the pathogenesis of lung diseases^54,55^. ECM stability should be therefore improved without MMP inhibitors. In addition, we only tested one donor of HBEC for this proof-of-concept study. As we reported previously, response to stimuli could vary depending on the donor characteristics^56,57^. Therefore, using multiple donors for toxicological and pharmacological testing with this BoC is warranted. Visualization of the BoC is also challenging issue because unlike 3D planer HBEC, cells in the BoC is aligned almost vertically. Laser-scanning confocal microscopy could be one of the options to capture the fluorescent images of the BoC, because of its high spatial resolution which enables us depth-wise imaging. The integrity of a barrier in the BoC is assessed through a single timepoint fluorescent measurement. Alternatively, the use of TransEpithelial Electrical Resistance (TEER) to assess the HBEC barrier has been explored as a more accurate method of assessing the barrier integrity. While further optimization is required to enhance the robustness of these measurements, they offer the potential for a more quantitative evaluation of barrier integrity in future studies. Assessment of the cilia function is also a challenging issue, because the visibility of beating cilia is very limited. Although the difficulty for time-course experiment due to such a limited visibility should be overcome, fluorescence imaging could be rather easier, therefore assessment method of ciliary function should be replaced with fluorescent beads-based method^58^. In addition, assessing ciliary function with high-speed camera and a specific software (e.g., SAVA system^59^) could be an alternative way, would thus be further investigated. In addition, an appropriate exposure apparatus compatible with the microfluidic device should be developed for controlled airway exposure inside the BoC.

## Conclusions

The novel microfluidic system BoC enabled a high-throughput OoC with an air-lifted tubular cell layer. The BoC contained three types of differentiated cells (basal, ciliated, and secretory cells), and thus has similar cell properties as human bronchi. In addition, EGF and IL-13, which are known inducers of airway epithelial remodeling in vitro and in vivo, induced a chronic response of mucus hypersecretion. These results show that this model can reproduce disease-state tissue characteristics through chronic exposure to known inducers and can be used as a high-function and high-throughput OoC assessment platform. As a next step, other toxicological (acute) assessments will be conducted with the BoC via medium exposure. Furthermore, because aerosols can pass through the BoC tube, a direct aerosol exposure assessment will be conducted with the development of an exposure device.

## Supplementary Information

Supplementary Material 1.

## Data Availability

No datasets were generated or analysed during the current study.
